# Long non-coding RNAs in neurodegenerative diseases—Molecular mechanisms, liquid biopsy biomarkers, and therapeutic targets: A review

**DOI:** 10.17305/bb.2026.13978

**Published:** 2026-03-31

**Authors:** Yipeng Cheng, Mingyue Qiu, Zhijie Yu, Xu Tang, Jingjing Zhang

**Affiliations:** 1 Chifeng Key Laboratory of Critical Illness and Complex Rare Diseases, Chifeng Municipal Hospital, Chifeng, China; 2 Chifeng Clinical Medical College of Inner Mongolia Medical University, Chifeng, China

**Keywords:** Long non-coding RNA, neurodegenerative diseases, liquid–liquid phase separation, ferroptosis, neuroinflammation, liquid biopsy, therapeutic targets

## Abstract

Neurodegenerative diseases (NDDs), such as Alzheimer’s disease (AD), Parkinson’s disease (PD), amyotrophic lateral sclerosis (ALS), and Huntington’s disease (HD), are age-related disorders characterized by progressive neuronal loss, cognitive decline, and limited options for disease-modifying treatments. Increasing evidence suggests that long non-coding RNAs (lncRNAs) play significant roles in neurodevelopment, neuronal homeostasis, and disease progression; however, their involvement in shared pathogenic pathways and clinical applications remains inadequately defined. This review consolidates recent experimental, transcriptomic, bioinformatic, and emerging clinical findings regarding the role of lncRNAs in NDDs. We examine how lncRNAs modulate common disease mechanisms, including protein misfolding and aggregation, neuroinflammation, mitochondrial dysfunction, ferroptosis, synaptic failure, and aging-related neurodegenerative processes. These regulatory functions occur through various mechanisms, including epigenetic modifications, transcriptional regulation, post-transcriptional processes, and RNA–protein interactions, as well as novel mechanisms such as liquid–liquid phase separation (LLPS), peptide coding, and exosome-mediated intercellular communication. Current evidence supports the potential of lncRNAs as minimally invasive liquid biopsy biomarkers, detectable in blood, cerebrospinal fluid (CSF), and extracellular vesicles. Additionally, lncRNAs may serve as therapeutic targets through antisense oligonucleotides (ASOs), gene editing, and engineered delivery platforms. Overall, lncRNAs have emerged as central molecular regulators and promising candidates for translation in NDDs. Nonetheless, challenges related to specificity, validation, delivery across the blood–brain barrier, and clinical standardization must be addressed before their routine application in precision neurology.

## Introduction

With the accelerated aging of the global population, the incidence rates of neurodegenerative diseases (NDDs) such as Alzheimer’s disease (AD), Parkinson’s disease (PD), amyotrophic lateral sclerosis (ALS), and Huntington’s disease (HD) continue to rise, presenting a significant public health challenge that threatens the health and quality of life of the elderly [1, 2]. Although the clinical phenotypes of various NDDs differ, they share common pathological features, including progressive neuronal death and irreversible functional decline. Currently, available clinical drugs provide only temporary symptomatic relief and do not reverse the progression of these diseases [3]. The core bottlenecks in addressing NDDs include: first, the lack of early diagnostic biomarkers, as many patients experience severe neuronal loss and brain structural damage by the time of diagnosis; second, the incomplete elucidation of pathogenic mechanisms, making single-target approaches inadequate to address the complexity and heterogeneity of these diseases; and third, the extremely low success rate of drug development. Therefore, there is an urgent need to explore novel intervention strategies. Increasing evidence suggests that NDDs are not solely caused by mutations in coding genes or protein abnormalities, but are complex disorders driven by dysregulation of the transcriptome, abnormal RNA metabolism, and disrupted cellular homeostasis.

Long non-coding RNAs (lncRNAs) are a class of transcripts longer than 200 nucleotides that do not encode proteins or only encode short peptides [4]. With advances in high-throughput sequencing technology, researchers have discovered that lncRNAs exhibit spatiotemporal specificity and cell subtype specificity. They are particularly abundant in the nervous system and are involved in critical biological processes, including neurogenesis [5], neuronal migration [6], synapse formation [7] and plasticity, and glial cell activation [8]. The classical regulatory mechanisms of lncRNAs have become relatively well-established. Recent studies have revealed advanced modes of lncRNA regulation, including mediating liquid–liquid phase separation (LLPS), encoding peptides [9], localizing to mitochondria to regulate energy metabolism, and facilitating intercellular communication via exosomes. These findings have greatly expanded our understanding of lncRNA functions and offered new perspectives for addressing NDDs.

## Overview of lncRNAs

The first abundantly expressed lncRNA in humans was discovered in 1990; it possesses a structure similar to that of messenger RNA (mRNA) but lacks coding potential [10]. However, progress in the field of lncRNAs remained relatively slow during the early stages due to the lack of precise detection technologies. It was not until 2002, when the sequencing of mouse complementary DNA (cDNA) libraries revealed tens of thousands of novel non-coding transcripts, that the identification and functional characterization of lncRNAs significantly accelerated [11]. To date, the GENCODE database has annotated more than 20,000 human lncRNAs, while other databases estimate that the number could exceed 100,000 [12, 13]. LncRNAs can be categorized based on their positional relationship with protein-coding genes:

- **Intergenic and Intronic lncRNA:** A substantial number of non-coding sequences are distributed between protein-coding genes [14]. LncRNAs transcribed from these intergenic regions are termed long intergenic non-coding RNAs (lincRNAs), while those transcribed entirely from the intronic regions of protein-coding genes are known as intronic lncRNAs.
- **Antisense lncRNA:** These are transcribed from the antisense strand of a protein-coding gene locus, with their sequence partially or completely complementary to the corresponding sense transcript [15].
- **Sense lncRNA:** These are transcribed from the sense strand of a protein-coding gene and overlap in genomic position with one or more exons of that gene [16].
- **Enhancer lncRNA:** These are transcribed from the enhancer regions of protein-coding genes [17].
- **Bidirectional lncRNA:** These are transcribed from an independent promoter located adjacent to a protein-coding gene promoter but in the opposite orientation [18].

LncRNAs possess complex secondary structures that enable them to interact with biomacromolecules, such as proteins, DNA, and RNA, thereby exerting their biological functions. The mechanisms of lncRNAs can be mainly divided into four categories [19, 20]:

- **Signal:** Serving as molecular signals that reflect and participate in specific cellular processes [15].
- **Decoy:** Binding to DNA-binding proteins or transcription factors to modulate downstream gene expression [21].
- **Guide:** Associating with chromatin-modifying complexes to form ribonucleoprotein complexes [21].
- **Scaffold:** Serving as structural platforms to assemble two or more proteins into a functional complex [21].

## Novel regulatory mechanisms of lncRNAs in NDDs

### Classical regulatory mechanisms

The classical regulatory mechanisms of lncRNAs are relatively well-established, providing a foundation for their function in the nervous system. At the epigenetic level, lncRNAs recruit histone modification enzymes [22], DNA methyltransferases [23], or chromatin remodeling complexes [24] to silence or activate specific genes. At the transcriptional level, lncRNAs can bind directly to transcription factors [25] or promoter [26] regions to regulate mRNA. At the post-transcriptional level, the primary function of lncRNAs involves the competing endogenous RNA (ceRNA) mechanism [27], which alleviates the inhibitory effects of microRNAs on target mRNAs by sequestering them. Furthermore, lncRNAs regulate mRNA splicing [28], stability [29], nuclear retention [30] and translation [31]. Although classical mechanisms can explain some functions of lncRNAs, they are insufficient to elucidate their spatial-specific and cell-specific roles in complex neuropathologies. Recent studies have revealed intricate regulatory mechanisms of lncRNAs, providing important theoretical insights into the occurrence and progression of NDDs (Figure 1).

**Figure 1.**
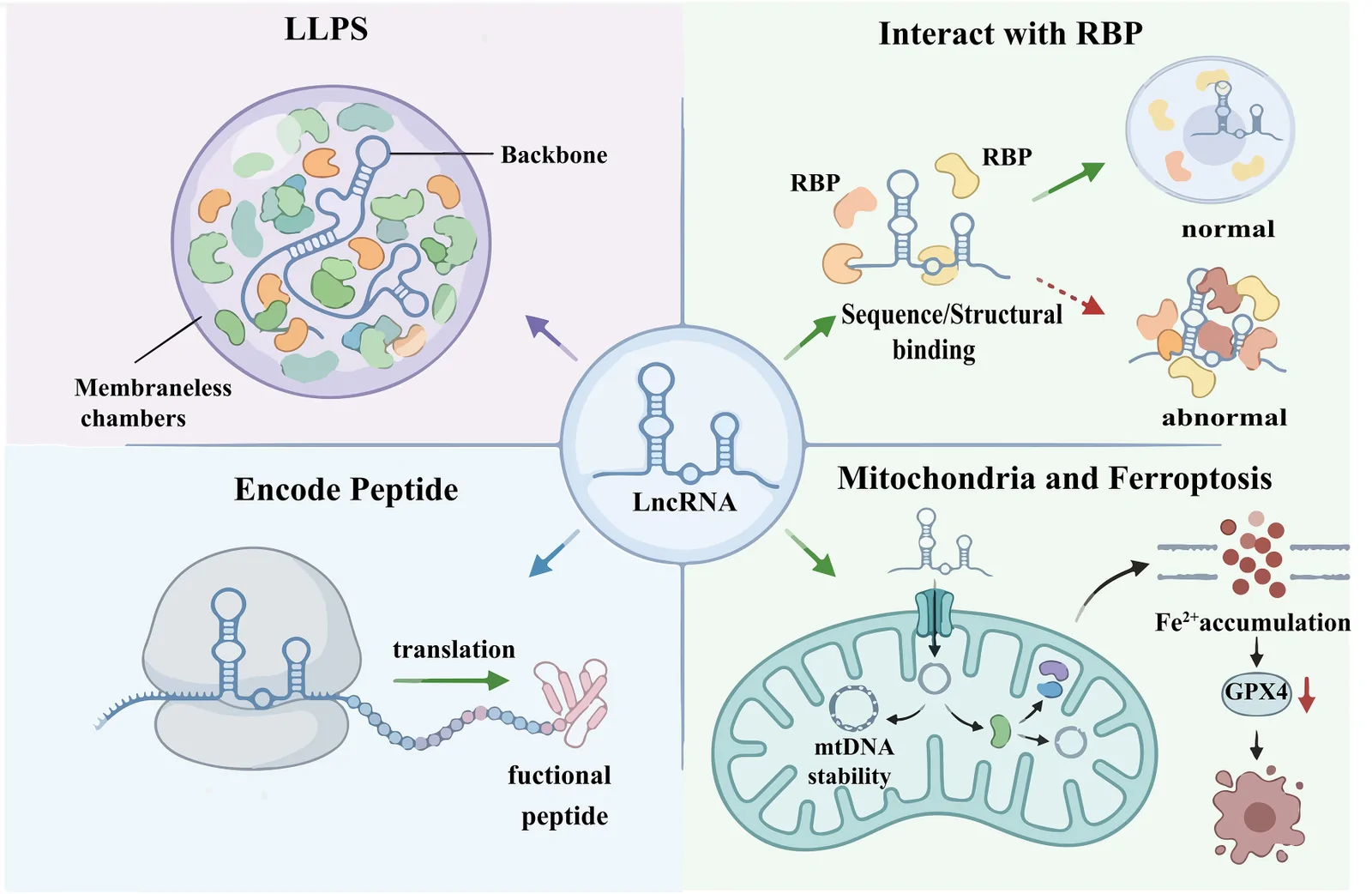
**Novel regulatory mechanisms of long non-coding RNAs in neurodegenerative diseases.** Schematic illustration of emerging regulatory mechanisms of long non-coding RNAs beyond their classical epigenetic, transcriptional, and post-transcriptional roles. Long non-coding RNAs may promote liquid–liquid phase separation by serving as structural backbones for ribonucleoprotein condensates and membraneless compartments; interact with RNA-binding proteins through sequence- or structure-dependent binding, thereby supporting either normal homeostasis or pathological protein dysregulation; encode functional peptides through translation of selected transcripts; and regulate mitochondrial function and ferroptosis by affecting mitochondrial DNA stability, iron accumulation, and glutathione peroxidase 4-associated antioxidant defense. Together, these mechanisms highlight the broad contribution of long non-coding RNAs to neurodegenerative disease pathogenesis. **Abbreviations:** Fe^2+^, ferrous iron; GPX4, glutathione peroxidase 4; LLPS, liquid–liquid phase separation; lncRNA, long non-coding RNA; mtDNA, mitochondrial DNA; RBP, RNA-binding protein.

### lncRNAs and LLPS

LLPS is an emerging biophysical phenomenon that can sequester molecules to implement physiological and pathological functions. LLPS facilitates the assembly of numerous membraneless compartments, including stress granules and P-bodies, which contain RNA and protein [32]. LncRNAs can act as backbone molecules that promote the phase separation of RNA-protein complexes due to their long chain structure, repeated sequences, and secondary structure. LLPS plays a fundamental role in various biological functions, including chromatin aggregation and cellular signal transduction [33]. Studies have indicated that lncRNAs capable of binding phospholipids [34] and participating in LLPS are involved in AD pathogenesis. However, few studies have directly investigated the regulation of phase separation by lncRNAs in NDDs, although some mechanistic insights have provided foundational support. For example, the lncRNA maternally expressed gene 3 (*MEG3*) modulates metabolism and muscle homeostasis by facilitating the LLPS of SUZ12 polycomb repressive complex 2 subunit (SUZ12), a core component of the polycomb repressive complex 2 (PRC2) [35]. These findings suggest that lncRNAs may function as scaffolds for LLPS, modulating the spatial localization and stability of protein complexes. This mechanism may also influence the epigenetics of the nervous system, thereby affecting neuronal epigenetic and stress responses through the regulation of LLPS.

### lncRNAs interact with RNA-binding proteins

The interaction between lncRNAs and RNA-binding proteins (RBPs) represents a fundamental regulatory mechanism in neurodegenerative diseases (NDDs). Abnormalities in nucleocytoplasmic transport, misfolding, and aggregation of RBPs—such as transactive response DNA-binding protein 43 kDa (TDP-43), tau, α-synuclein, and fused in sarcoma (FUS)—are critical pathological hallmarks of NDDs. LncRNAs can interact with RBPs through sequence complementarity or structural binding, thereby maintaining normal localization and function or exacerbating abnormal aggregation under pathological conditions [36]. Studies have found that the lncRNA family with sequence similarity 151 member B divergent transcript (*FAM151B-DT*) binds to tau protein, α-synuclein, and heat shock cognate 70 kDa protein (HSC70) to form a complex that regulates the autophagic degradation of pathogenic proteins [37]. LncRNAs maintain neuronal homeostasis by regulating RBPs and participating in various biological processes, such as RNA splicing, localization, and degradation [38], which can lead to widespread imbalances in post-transcriptional regulation [39]. Additionally, regulatory relationships between lncRNAs and RBPs also influence aging-related NDDs [40]. Furthermore, lncRNAs can modulate signal transduction within the nervous system by establishing competitive regulatory networks with RBPs [41]. The specificity of interactions between lncRNAs and RBPs provides an important basis for understanding the molecular mechanisms underlying the early stages of NDDs.

### lncRNAs encoding peptides

Over the past decade, traditional views have held that lncRNAs lack protein-coding ability. However, a significant body of research has demonstrated the existence of functional peptides translated from lncRNAs [42]. Currently, the peptide-coding function of various lncRNAs has been confirmed in pathological processes such as cancer, metabolic disorders, and inflammatory responses [43, 44], providing critical insights for investigating similar mechanisms in NDDs. Studies indicate that peptides play a role in the onset and progression of diseases by regulating key pathways, including protein homeostasis, mitochondrial function, neuroinflammation, and synaptic function [45].

For instance, lncRNA *1810058I24Rik* encodes a mitochondrion-localized peptide, Stmp1, which is highly expressed in retinal ischemia-reperfusion (IR) mouse models. This peptide exacerbates microglial activation and neuroinflammation by triggering the NLR family pyrin domain containing 3 (NLRP3) inflammasome [46]. Additionally, lncRNA *MN3* encodes a peptide, SERTM2, specifically enriched in spinal cord motor neurons. Both wild-type and SERTM2 knockout motor neurons, differentiated *in vitro* from mouse embryonic stem cells, exhibit differences in resting membrane potential and an overall decrease in excitability upon SERTM2 depletion. *In vivo* studies indicate that the absence of this peptide impairs performance in treadmill tests and modulates motor neuron excitability through interaction with the TASK1 potassium channel [47]. Furthermore, lncRNA potassium voltage-gated channel subfamily Q member 1 opposite strand/antisense transcript 1 (*KCNQ1OT1*) exacerbates inflammatory responses and promotes dopaminergic neuron death in PD by regulating the *miR-186/*NLRP3 axis and the NLRP3 inflammasome [48]. LncRNA PR/SET domain 16 divergent transcript (*PRDM16-DT*) has been identified as an astrocyte-specific lncRNA in AD, participating in the disease’s pathological processes by affecting synapse formation [49, 50].

Although direct evidence of lncRNAs being translated into peptides is currently lacking, these transcripts may possess undiscovered peptide-coding potential. Moreover, studies suggest that different splice isoforms may confer distinct subcellular localization and translational potential to lncRNAs [51]. For example, lncRNA *3222401L13Rik* is upregulated in aging astrocytes and affects neuronal protective functions by regulating Neuronal PAS domain protein 3 (Npas3) [52]. This dynamic expression pattern provides a crucial foundation for further investigation into the peptide-encoding mechanisms of lncRNAs.

### lncRNAs in mitochondrial dysfunction and ferroptosis

Mitochondrial dysfunction is a prevalent early pathological characteristic in NDDs [53]. Recent studies have identified a class of lncRNAs localized in mitochondria that can directly regulate mitochondrial DNA (mtDNA) stability [54], respiratory chain efficiency [55], mitophagy [56], and mitochondrial fission and fusion [57]. For instance, lncRNA *lncMtDloop*, encoded by the D-loop region of the mitochondrial genome, maintains mitochondrial RNA levels and function during aging. Research has demonstrated that *lncMtDloop* enhances the transcription of mitochondrial genes by binding to mitochondrial transcription factor A (TFAM), facilitating its recruitment to the promoter regions of mitochondrial DNA. Additionally, the expression of *lncMtDloop* is significantly downregulated in the brain tissues of AD patients and triple-transgenic Alzheimer’s disease (3xTg-AD) mouse models. Ectopic expression of *lncMtDloop* not only significantly improves mitochondrial function and morphology but also effectively alleviates behavioral deficits in AD model mice [58]. The lncRNA *LOC339803* regulates glycolysis and mitochondrial energy metabolism to preserve mitochondrial integrity by maintaining hexokinase 2 (HK2) expression levels in neuronal cells. Abnormal expression of *LOC339803* has been observed in the brain tissues of multiple sclerosis (MS) patients, leading to HK2 downregulation and mitochondrial dysfunction [59]. The lncRNA*ROSALIND*, encoded by the nuclear genome and localized to the mitochondrial matrix, is significantly upregulated in recurrent tumors. Studies indicate that *ROSALIND* protects the mitochondrial translation process from oxidative stress-induced damage, while its downregulation can cause severe defects in mitochondrial respiratory function [60].

Furthermore, mitochondrial lncRNAs are implicated in regulating ferroptosis, a type of programmed cell death characterized by intracellular iron accumulation, lipid peroxidation, downregulation of the antioxidant enzyme glutathione peroxidase 4 (GPX4), and mitochondrial membrane ruffling [61]. In ALS lesions, significant iron accumulation occurs in the spinal cord [62]. For example, lncRNA *LINC00472* is upregulated in both AD patients and shows a positive correlation with forkhead box O1 (FOXO1). Knockdown of *LINC00472* reduces abnormal iron ion accumulation in neurons and restores GPX4 expression by regulating FOXO1. Research indicates that *LINC00472* mediates cellular ferroptosis by targeting FOXO1, contributing to AD regulation [63]. In recent years, an increasing number of studies have linked mitochondrial lncRNA-mediated RNA regulation, energy metabolism, and oxidative stress processes with ferroptosis, indicating promising research directions for the future.

## lncRNAs mediate common pathological pathways in neurodegenerative diseases

Despite the distinct clinical and pathological features of NDDs, research has shown that these disorders share a series of common dysregulated pathways at the cellular and molecular levels. LncRNAs play a central role in the dysregulation of these shared pathological pathways (Figure 2).

**Figure 2.**
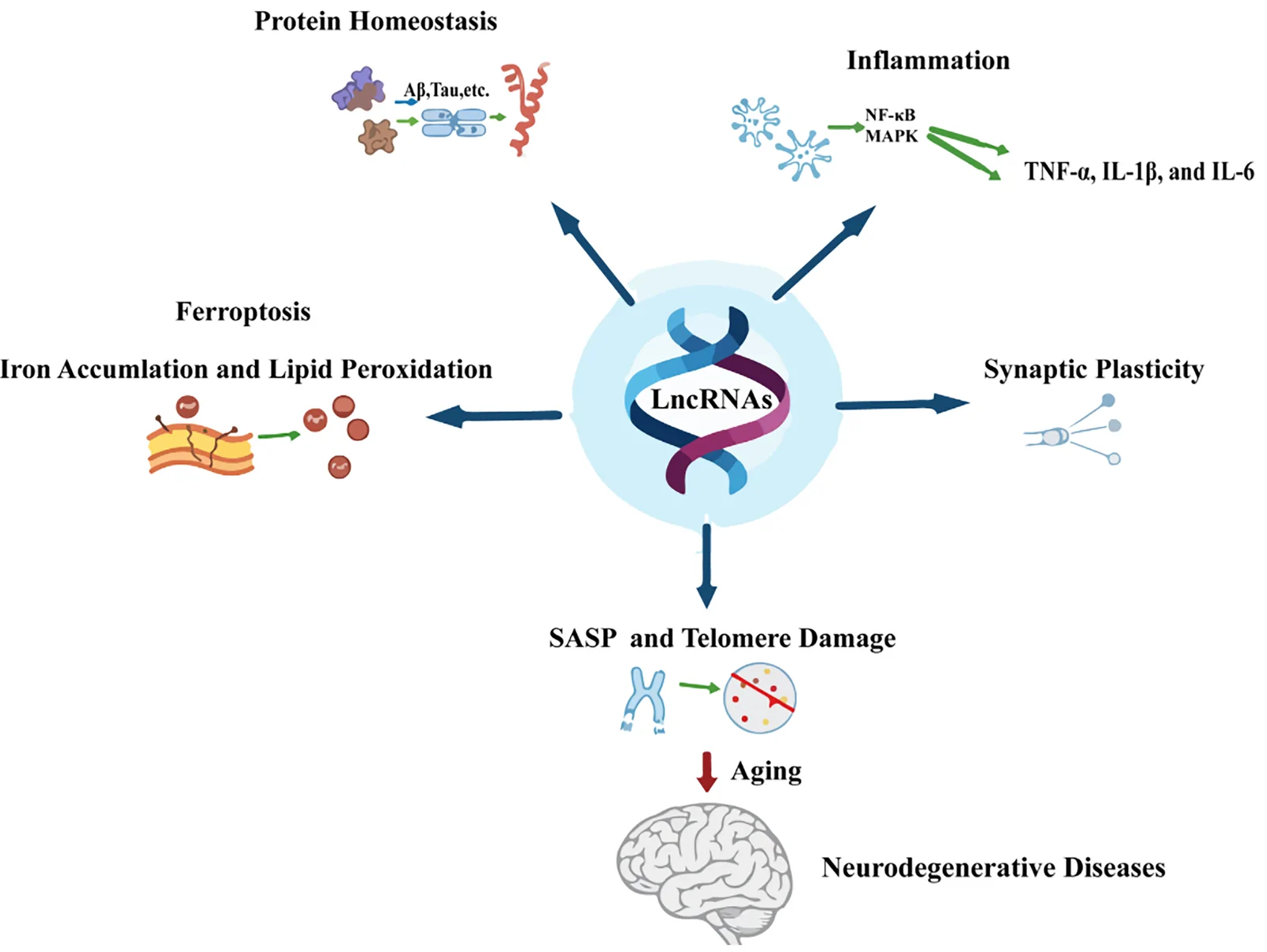
**Long non-coding RNAs mediate common pathological pathways in neurodegenerative diseases.** Schematic overview of the major shared pathological pathways regulated by long non-coding RNAs across neurodegenerative diseases. Although individual neurodegenerative disorders differ in clinical presentation, long non-coding RNAs converge on common mechanisms, including disruption of protein homeostasis, activation of inflammatory pathways, impairment of synaptic plasticity, ferroptosis characterized by iron accumulation and lipid peroxidation, and aging-related changes associated with the senescence-associated secretory phenotype and telomere damage. Through these interconnected processes, long non-coding RNAs contribute to neuronal dysfunction and disease progression. **Abbreviations:** Aβ, amyloid beta; IL-1β, interleukin 1 beta; IL-6, interleukin 6; lncRNA, long non-coding RNA; MAPK, mitogen-activated protein kinase; NF-κB, nuclear factor kappa B; SASP, senescence-associated secretory phenotype; Tau, tau protein; TNF-α, tumor necrosis factor alpha.

### lncRNAs and protein homeostasis

Protein misfolding and abnormal aggregation represent core pathological mechanisms across all NDDs. Amyloid-beta (Aβ) and tau proteins are the principal abnormally aggregated proteins, with their pathological alterations serving as primary drivers of AD. Aβ is generated from amyloid precursor protein through sequential cleavage by β-secretase and γ-secretase, with aggregates triggering neuroinflammation [64]. Tau, a microtubule-associated protein localized within neurons, stabilizes microtubule structures and facilitates axonal transport. Abnormal aggregation of tau exhibits neurotoxicity and disrupts organelle functions [64]. Studies have indicated that lncRNAs regulate key pathogenic proteins at multiple levels (Table 1).

**Table 1:** Regulation of pathogenic proteins by lncRNAs in neurodegenerative diseases

**LncRNA**	**NDDs**	**Changes**	**Target protein(s)**	**Function**	**Reference**
BACE1-AS	AD	Up in AD patients and AD cell model	Aβ	Down-regulation of BACE1-AS can reduce the production of Aβ_1−__4__2_ and Aβ_1−__4__0_, and delay amyloid plaque formation.	[66]
MEG3	AD	Down in AD rat model	Aβ	Upregulation of MEG3 expression reduces Aβ deposition in hippocampal tissue.	[67]
lncRNA 17A	AD	Up in AD patients and AD cell model	Aβ	Down-regulated promote Aβ secretion.	[68]
lncRNA EBF3-AS	AD	Up in APP/PS1 mouse	Aβ	Down-regulated inhibit Aβ_2__5__−__3__5_-induced apoptosis.	[69]
lncRNA BC1	AD	Up in Tg2576-APPswe AD mouse model	Aβ	Down-regulation aimed at enhancing cognitive function by disrupting the interaction between BC1 and FMRP can inhibit APP translation and Aβ deposition.	[70]
NEAT1	AD	Up in AD cell model	Aβ/tau	Down-regulated to alleviate Aβ-induced inhibition of cell viability, reduce apoptosis, and decrease p-Tau protein levels.	[71]
FAM151B-DT	AD/PD	Up in PD mouse model and MPP^+^ SK-N-SH / MN9D cells	Tau/α-syn	Down-regulated to mitigate the aggregation of aberrant proteins, this process involves interactions with HSC70, Tau protein, and α-synuclein to promote autophagic clearance.	[37]
lncRNA PINK1	PD	Up in PD mouse model	PINK1	Indirectly affect the aggregation and clearance of α-syn by regulating mitophagy-related proteins.	[72]
NEAT1	PD	Up in PD mouse model and PD cell model	PINK1/α-syn	Inhibits the degradation of the PINK1 protein and positively regulates its levels. Additionally, it is downregulated to mitigate the activation of the NLRP3 inflammasome induced by α-syn.	[73, 74]
UCA1	PD	Up in PD mouse model and PD cell model	α-syn	Upregulation of UCA1 enhances the mRNA and protein levels of α-synuclein, while its knockdown inhibits neuronal apoptosis.	[75]
BACE1-AS	PD	Up in PD rat model	α-syn	Down-regulation inhibits Aβ_2__5__−__3__5_-induced apoptosis.	[76]
NEAT1-2	ALS	Up in ALS patients	TDP-43	NEAT1-2 is induced by the nuclear clearance of TDP-43 and drives the formation of paraspeckles. Paraspeckles may exert an early protective effect by sequestering aberrant RNAs or proteins.	[78]
HTTA-v1	HD	Down in HD patients	HTT	Negatively regulates HTT expression.	[79]

**Abbreviations:** Aβ, amyloid beta; AD, Alzheimer’s disease; ALS, amyotrophic lateral sclerosis; APP, amyloid precursor protein; BACE1-AS, BACE1 antisense RNA; FMRP, fragile X mental retardation protein; HD, Huntington’s disease; HSC70, heat shock cognate 70; HTT, huntingtin; HTTAS-v1, huntingtin antisense splice variant 1; lncRNA, long non-coding RNA; MEG3, maternally expressed 3; MPP^+^, 1-methyl-4-phenylpyridinium; NDDs, neurodegenerative diseases; NEAT1, nuclear paraspeckle assembly transcript 1; NEAT1-2, nuclear paraspeckle assembly transcript 1 isoform 2; NLRP3, NLR family pyrin domain containing 3; PD, Parkinson’s disease; PINK1, PTEN-induced kinase 1; Tau, tau protein; UCA1, urothelial cancer associated 1; α-syn, alpha-synuclein.

LncRNA beta-site APP-cleaving enzyme 1 antisense transcript (*BACE1-AS*) is significantly upregulated in the brains of AD patients. It has been found to enhance β-secretase activity and promote Aβ production by binding to the sense strand of beta-site APP-cleaving enzyme 1 (*BACE1*) mRNA, stabilizing it and enhancing protein translation [65]. Similarly, *BACE1-AS* was upregulated in human neuroblastoma SH-SY5Y cells treated with Aβ_1−__4__2_, establishing an *in vitro* model of AD amyloid plaques. Silencing *BACE1-AS* attenuated the cleavage of amyloid precursor protein (APP) by *BACE1*, reducing the generation of Aβ_1−__4__2_ and Aβ_1−__4__0_, consequently delaying amyloid plaque formation. This study indicates that lncRNA *BACE1-AS* is a critical factor in Aβ_1−__4__2_ formation [66].

LncRNA *MEG3* was significantly downregulated in an Aβ_2__5__−__3__5_-induced AD rat model. Upregulation of MEG3 enhanced spatial learning and memory in rats, decreased Aβ deposition in the hippocampus, and mitigated oxidative stress and inflammatory damage by inhibiting the phosphatidylinositol 3-kinase (PI3K)/protein kinase B (Akt) signaling pathway. This study suggests that lncRNA *MEG3* is a key regulatory factor in Aβ formation [67].

Another study found that lncRNA *17A* was significantly upregulated in the brains of AD patients and SH-SY5Y neuroblastoma cells, with overexpression of lncRNA *17A* promoting Aβ secretion and increasing the Aβ_x−__4__2_/Aβ_x−__4__0_ ratio. This study confirms that lncRNA *17A* is involved in the pathological progression of AD by regulating Aβ secretion [68].

LncRNA *EBF3-AS* was significantly upregulated in the hippocampus of amyloid precursor protein / presenilin 1 (*APP/PS1*) AD mice. Knockdown of lncRNA early B-cell factor 3 antisense RNA (*EBF3-AS*) inhibited Aβ_2__5__−__3__5_-induced apoptosis, indicating that lncRNA *EBF3-AS* promotes neuronal apoptosis in AD [69].

LncRNA brain cytoplasmic RNA 1 (*BC1*) was aberrantly upregulated in the brains of AD mice. It binds to Fragile X Mental Retardation Protein 1 (FMRP), relieving the translational repression of APP mRNA by FMRP, thereby promoting the generation and aggregation of Aβ. Inhibiting*BC1* expression improved cognitive function by disrupting Aβ deposition, indicating that lncRNA *BC1* influences Aβ production by regulating FMRP-mediated APP translation and participates in the pathological progression of AD [70].

LncRNA nuclear paraspeckle assembly transcript 1 (*NEAT1*) was significantly upregulated in an AD cell model established by Aβ induction in SH-SY5Y cells. Knockdown of *NEAT1* alleviated Aβ-induced inhibition of cell viability, reduced apoptosis, and decreased phosphorylated Tau (p-Tau) protein levels. Furthermore, *miR-107* was significantly decreased in Aβ-treated SH-SY5Y cells, while overexpression of *miR-107* could reverse Aβ-induced cellular damage. This study indicates that *NEAT1* exacerbates Aβ-induced neuronal damage by competitively binding to*miR-107* [71].

LncRNA *FAM151B-DT* was significantly downregulated in the brains of AD and PD patients. Knockdown of *FAM151B-DT* inhibited autophagy and promoted the aggregation of tau protein and α-synuclein, whereas overexpression of *FAM151B-DT* enhanced autophagic clearance by interacting with HSC70, tau protein, and α-synuclein, significantly reducing the aggregation of abnormal proteins [37].

α-Synuclein (α-syn) is a core pathogenic protein, and its abnormal aggregation is a hallmark of PD. Additionally, dysfunction of associated pathogenic proteins, such as leucine-rich repeat kinase 2 (LRRK2), Parkin, PTEN-induced kinase 1 (PINK1), and glucocerebrosidase (GBA), contributes to impaired clearance of α-syn and exacerbates its aggregation, ultimately leading to selective neuronal loss.

A study has demonstrated that an antisense lncRNA upstream of *PINK1* inhibits its transcription. The *PINK1* protein plays a crucial role in the clearance of damaged mitochondria and is associated with α-syn aggregation. This research suggests that lncRNAs may indirectly influence the aggregation and clearance of α-syn by regulating mitophagy-related proteins [72].

The lncRNA *NEAT1* is upregulated in a PD mouse model. It positively regulates *PINK1* protein levels by inhibiting its degradation. Conversely, the knockdown of *NEAT1* effectively inhibits 1-methyl-4-phenyl-1,2,3,6-tetrahydropyridine (MPTP)-induced autophagy *in vivo*, thereby alleviating damage to dopaminergic neurons. This finding indicates that *NEAT1* promotes MPTP-induced autophagy in the PD model by stabilizing the PINK1 protein [73]. Furthermore, *NEAT1* is upregulated in 1-methyl-4-phenylpyridinium (MPP^+^) SH-SY5Y cells, and its downregulation alleviates α-syn-induced NLRP3 inflammasome activation by regulating miR-1301-3p [74].

The lncRNA urothelial carcinoma associated 1 (*UCA1*) has been implicated in the progression of PD by regulating the expression of α-syn. Both *UCA1* and α-syn are highly expressed in the brain tissues of PD mouse models and in MPP^+^ SH-SY5Y cells. Knockdown of *UCA1* effectively inhibits MPP^+^-induced neuronal apoptosis by preventing α-syn aggregation [75].

The lncRNA *BACE1-AS* is upregulated in the substantia nigra of PD rat models. Downregulation of *BACE1-AS* reduces levels of α-syn, inducible nitric oxide synthase, and glutamine, indicating that *BACE1-AS* mediates oxidative stress injury in dopaminergic neurons by regulating α-syn [76].

In ALS and HD, pathogenic proteins, including superoxide dismutase 1 (SOD1), FUS, chromosome 9 open reading frame 72 (C9orf72), and TDP-43, are primarily RNA-binding proteins. HD arises from a monogenic dominant mutation in huntingtin (HTT), with its pathogenic mechanism linked to the intrinsic toxicity of the mutant protein [77]. Studies have revealed that lncRNAs influence the toxicity of mutant TDP-43, FUS, HTT, SOD1, and C9ORF72. The lncRNA *NEAT1-2* is significantly upregulated in motor neurons during the early pathological stages of ALS, induced by the nuclear clearance of TDP-43, suggesting that lncRNAs directly respond to TDP-43 pathological changes [78]. Additionally, huntingtin antisense transcript variant 1 (*HTTAS-v1*) is significantly downregulated compared to controls in HD patients. It negatively regulates HTT gene expression in Dicer-knockout mouse embryonic stem cells, indicating that *HTTAS-v1* is a key regulator of HTT [79].

### lncRNAs and neuroinflammation

Neuroinflammation is a central factor in the progression of NDDs. Aberrant activation of classical inflammatory signaling pathways, such as nuclear factor kappa-light-chain-enhancer of activated B cells (NF-κB) and mitogen-activated protein kinase (MAPK), is crucial for neuronal damage. The overactivation of microglia and astrocytes amplifies inflammation by releasing large amounts of pro-inflammatory factors. Studies indicate that lncRNAs can regulate glial cell activation and polarization, as well as the release of inflammatory factors such as tumor necrosis factor alpha (TNF-α), interleukin (IL)-1β, and IL-6, by targeting inflammation-related signaling pathways, including NF-κB, MAPK, and signal transducer and activator of transcription 3 (STAT3), or through ceRNA networks and epigenetic modulation. This regulation influences the pathological progression of neuroinflammation (Table 2) [80].

**Table 2:** lncRNAs mediating inflammatory responses in neurodegenerative diseases

**LncRNA**	**NDDs**	**Changes**	**Function**	**Reference**
SNHG14	AD	Up in AD patients; Up in APP/PS1 mice	Binds MRTFA and MRTFB, resulting in an interaction between neurons and glial cells; the sponge of miR-223-3p suppresses astrocytic NLRP3 inflammasome-mediated neuroinflammation.	[81, 83]
MALAT1	AD	Down in AD cell model	Upregulated to inhibit neuronal apoptosis and promote neurite outgrowth, the sponge of miR-125b reduces the levels of IL-6 and TNF-α.	[82]
MEG3	AD	Down in AD mouse model	Up-regulated to alleviate inflammatory injury by modulating the PI3K/Akt signaling pathway.	[67]
PRR34-AS1	AD	Up in AD patients and AD cell mode	Down-regulation alleviates microglia-mediated inflammatory responses by upregulating miR-29c-3p expression.	[84]
NEAT1	PD	Up in PD mouse model and MPP^+^ SH-SY5Y cells	Down-regulation suppresses MPTP-induced autophagy *in vivo*, thereby alleviating dopaminergic neuronal injury.	[73]
MALAT1	PD	Up in PD mouse model and cell model	Down-regulation reduces the methylation level of the SOCS3 promoter, thereby alleviating neuroinflammation and neuronal injury.	[85]
SOX21-AS1	PD	Up in PD mouse model	Down-regulation reduces microglial activation by upregulating SOCS3 expression, thereby alleviating neuroinflammation.	[86]

**Abbreviations:** AD, Alzheimer’s disease; APP/PS1, amyloid precursor protein/presenilin 1; Akt, protein kinase B; IL-6, interleukin 6; lncRNA, long non-coding RNA; MALAT1, metastasis associated lung adenocarcinoma transcript 1; MEG3, maternally expressed 3; miR, microRNA; MPP^+^, 1-methyl-4-phenylpyridinium; MPTP, 1-methyl-4-phenyl-1,2,3,6-tetrahydropyridine; MRTFA, myocardin related transcription factor A; MRTFB, myocardin related transcription factor B; NDDs, neurodegenerative diseases; NEAT1, nuclear paraspeckle assembly transcript 1; NLRP3, NLR family pyrin domain containing 3; PD, Parkinson’s disease; PI3K, phosphatidylinositol 3-kinase; PRR34-AS1, PRR34 antisense RNA 1; SH-SY5Y, human neuroblastoma cell line; SNHG14, small nucleolar RNA host gene 14; SOCS3, suppressor of cytokine signaling 3; SOX21-AS1, SOX21 antisense RNA 1; TNF-α, tumor necrosis factor alpha.

A subcluster of neurons and glial cells co-expressing lncRNA small nucleolar RNA host gene 14 (*SNHG14*), myocardin-related transcription factors A (MRTFA) and B (MRTFB) was found to be more abundant in the AD group based on single-nucleus RNA sequencing (snRNA-seq) data from Gene Expression Omnibus (GEO) datasets. This subcluster is enriched in MAPK, immune, and apoptosis-related pathways, suggesting that lncRNA *SNHG14* may interact with MRTFA and MRTFB, facilitating communication between neurons and glial cells [81].

The lncRNA metastasis associated lung adenocarcinoma transcript 1 (*MALAT1*) was significantly downregulated in AD models and nerve growth factor (NGF)-stimulated PC12 cells, while miR-125b was upregulated. Overexpression of lncRNA *MALAT1* inhibited neuronal apoptosis, promoted neurite outgrowth, reduced IL-6 and tumor necrosis factor alpha (TNF-α) levels, and increased IL-10 levels. Conversely, knockdown of *MALAT1* promoted neuronal apoptosis, repressed neurite outgrowth, elevated IL-6 and TNF-α levels, and reduced IL-10 levels. Additionally, *MALAT1* inversely regulated the expression of *miR-125b*. These findings suggest that lncRNA *MALAT1* may interact with *miR-125b* to inhibit neuronal apoptosis and inflammation while promoting neurite outgrowth in AD [82].

The lncRNA *SNHG14* is significantly upregulated in the *APP/PS1* mouse model of AD, correlating with increased levels of IL-1β, IL-6, and TNF-α, along with a significant downregulation of *miR-223-3p*. Moreover, AVE0991 (a non-peptide analogue of Ang-(1–7)) downregulates *SNHG14* expression, mitigating its sponging effect on *miR-223-3p* and blocking the neuroinflammatory response. This finding confirms that lncRNA *SNHG14* influences the neuroinflammatory response by modulating *the miR-223-3p/NLRP3 signaling pathway* [83].

The lncRNA *MEG3* is significantly downregulated in AD mouse models. Upregulation of *MEG3* alleviates inflammatory damage by modulating the PI3K/Akt signaling pathway, exerting neuroprotective effects [67].

The lncRNA PRR34 antisense RNA 1 (PRR34-AS1) is significantly upregulated in the serum of AD patients and in Aβ_2__5__−__3__5_-treated HMC3 and BV2 microglial AD models, while the expression of *miR-29c-3p* is significantly downregulated. PRR34-AS1 exhibits a negative correlation with the neuroinflammatory processes in AD. Knockdown of *PRR34-AS1* alleviates the aforementioned pathological damage by upregulating *miR-29c-3p* expression. The results suggest that the *PRR34-AS1/miR-29c-3p* signaling axis participates in the pathological progression of AD by modulating microglia-mediated inflammatory responses [84].

The lncRNA *NEAT1* is closely associated with the pathological progression of PD. In the MPP^+^-induced PD model, levels of pro-inflammatory factors such as TNF-α, IL-6, and IL-1β are significantly upregulated, indicating a relationship between *NEAT1* and the neuroinflammatory response [73].

The lncRNA *MALAT1* is upregulated in MPTP-induced PD mice and lipopolysaccharide (LPS)-induced BV2 cells. Silencing of *MALAT1* increases cell viability while inhibiting apoptosis and inflammation in LPS-induced BV2 cells. Additionally, *MALAT1* enhances the methylation of the suppressor of cytokine signaling 3 (SOCS3) promoter, decreasing its expression by recruiting DNA methyltransferase 1 (DNMT1), DNA methyltransferase 3 alpha (DNMT3A), and DNA methyltransferase 3 beta (DNMT3B). Furthermore, knockdown of SOCS3 eliminates the inhibition of LPS-induced BV2 cell injury mediated by sh-*MALAT1*. *In vivo*, silencing of *MALAT1* improves neurological impairment and neuroinflammation in MPTP-induced PD mice. These findings suggest that *MALAT1* exacerbates PD processes by inhibiting *SOCS3* expression through increased promoter methylation [85]. Additionally, the lncRNAs SOX21 antisense RNA 1 (*SOX21-AS1*) and *SOCS3* are upregulated in PD cell and mouse models. Inhibition of *SOX21-AS1* represses LPS/adenosine triphosphate (ATP)-induced activation in BV2 cells and mitigates nerve damage caused by activated BV2 cells, alleviating the pathological features of PD. *SOX21-AS1* epigenetically inhibits *SOCS3* by recruiting enhancer of zeste homolog 2 (EZH2) to its promoter. Overexpression of SOX21-AS1 partially offsets the repressive impact of *SOCS3* enhancement on BV2 cell activation and the protective effect on nerve cells. This study suggests that *SOX21-AS1* enhances LPS/ATP-induced BV2 cell activation and nerve damage caused by activated BV2 cells through its recruitment of EZH2 to the *SOCS3* promoter [86].

The lncRNA small nucleolar RNA host gene 1 (*SNHG1*) and mitogen-activated protein kinase 1 (MAPK1) are upregulated in the MPTP-induced PD mouse model. SNHG1 activates the MAPK signaling pathway, exacerbating neuronal apoptosis and oxidative stress by sponging *miR-125b-5p*, which alleviates inhibition of MAPK1. Conversely, knockdown of *SNHG1* or overexpression of *miR-125b-5p* inhibits MAPK1 expression and mitigates pathological damage. These results indicate that the *SNHG1/miR-125b-5p*/MAPK1 signaling axis may participate in PD by modulating neuroinflammation [87].

### lncRNAs and ferroptosis

Neuronal loss is a common endpoint in NDDs, but the mode of neuronal death is not exclusively apoptosis. Recent evidence confirms that ferroptosis drives early synaptic impairment and neuronal death in AD and PD. Ferroptosis is a novel form of cell death characterized by iron-dependent lipid peroxidation. Accumulating evidence indicates that various NDDs are prominently associated with localized iron accumulation in specific regions of the central or peripheral nervous system [88]. Moreover, studies have shown that ferroptosis is broadly implicated in multiple pathological events in PD, including abnormal α-syn aggregation, mitochondrial dysfunction, and neuroinflammation, all of which exhibit crosstalk with ferroptosis signaling pathways [89]. Further research demonstrates that lncRNAs play a crucial upstream regulatory role in ferroptosis pathways (Table 3). Studies have established a ferroptosis-related signature model, including mRNAs, microRNAs (miRNAs), and lncRNAs, and characterized its association with immune infiltration in AD [90, 91]. These findings offer new insights into the role of ferroptosis-related molecular patterns and immune mechanisms in AD, providing a theoretical basis for the identification of diagnostic markers for the disease.

**Table 3:** lncRNAs and ferroptosis in neurodegenerative diseases

**LncRNA**	**NDDs**	**Changes**	**Function**	**Reference**
HEIH, SNHG15, and SNHG5	AD/PD	Bioinformatics analysis of a new framework programmed cell death Lnc	These lncRNAs and their associated genes are involved in programmed cell death in neurodegenerative diseases, offering potential therapeutic targets and diagnostic markers for future study and clinical application.	[90]
TUG1	AD	Up in AD patients; and AD mouse/cell models	TUG1 silencing attenuated Aβ1–42-induced ferroptosis and neurotoxicity	[92]
LINC00472	AD	Up in AD mouse/cell model	Down-regulation inhibits ferroptosis by reducing iron accumulation.	[63]
NEAT1	PD	Up in PD mouse/cell model; Up in neurotoxic PD models; Up in AD patients; and AD mouse/cell model	Knockdown of NEAT1 mitigated MPP+-induced ferroptosis through regulating BAP1/SLC7A11 axis by sponging miR-150-5p; NEAT1-PIK3CA/ATM ceRNA network mediates α-syn-driven ferroptosis in PD; Down-regulated to blocks he ferroptosis capacities of cells.	[93–95]

**Abbreviations:** Aβ1–42, amyloid beta 1–42; AD, Alzheimer’s disease; ATM, ATM serine/threonine kinase; BAP1, BRCA1 associated deubiquitinase 1; ceRNA, competing endogenous RNA; HEIH, hepatocellular carcinoma up-regulated EZH2-associated long non-coding RNA; LINC00472, long intergenic non-protein coding RNA 472; lncRNA, long non-coding RNA; MPP^+^, 1-methyl-4-phenylpyridinium; NDDs, neurodegenerative diseases; NEAT1, nuclear paraspeckle assembly transcript 1; PD, Parkinson’s disease; PIK3CA, phosphatidylinositol-4,5-bisphosphate 3-kinase catalytic subunit alpha; SLC7A11, solute carrier family 7 member 11; SNHG5, small nucleolar RNA host gene 5; SNHG15, small nucleolar RNA host gene 15; TUG1, taurine up-regulated 1; α-syn, alpha-synuclein.

The lncRNA methyltransferase-like 14 (*METTL14*) is downregulated, while lncRNA taurine upregulated gene 1 (*TUG1*) is upregulated in clinical patients and experimental models of AD. Overexpression of *METTL14* or silencing of *TUG1* effectively attenuates Aβ1–42-induced ferroptosis and neurotoxicity in SH-SY5Y cells [92].

The lncRNA *LINC00472* is significantly upregulated in AD samples derived from GEO datasets and is positively correlated with *FOXO1* expression. Knockdown of either *LINC00472* or *FOXO1* inhibits ferroptosis by reducing iron accumulation and upregulating GPX4 expression in Aβ_2__5__−__3__5_-induced AD mouse models and HT22 cells. This study reveals that *LINC00472* plays a significant role in the regulation of ferroptosis in AD [63].

The lncRNA *NEAT1* is significantly upregulated in the MPP^+^-induced PD model of SK-N-SH cells. Knockdown of *NEAT1* enhances cell viability and glutathione (GSH) levels in the PD cell model while reducing levels of reactive oxygen species (ROS), malondialdehyde (MDA), and Fe^2+^. *NEAT1* functions as a sponge to suppress *miR-150-5p* expression, and overexpression of *miR-150-5p* suppresses ferroptosis in the PD cell model. This study suggests that knockdown of *NEAT1* mitigates MPP^+^-induced ferroptosis through sponging *miR-150-5p* [93].

Additionally, a study based on bioinformatics analysis of 97 human substantia nigra tissue samples (54 PD patients and 43 healthy controls) from the GEO database revealed that *NEAT1* was significantly upregulated across multiple cell types in the substantia nigra of PD patients. Moreover, PD-associated ferroptosis genes were significantly enriched in signaling pathways such as MAPK and calcium signaling, suggesting a close association with neuroinflammatory processes [94]. Similarly, a study investigating α-syn-driven ferroptosis in PD included 16 human substantia nigra tissue samples (8 PD patients and 8 healthy controls) from the GEO database. Bioinformatics analysis identified hub genes such as phosphatidylinositol-4,5-bisphosphate 3-kinase catalytic subunit alpha (*PIK3CA*), ataxia telangiectasia mutated (*ATM*), and bromodomain containing protein 4 (*BRD4*), as well as the key lncRNA *NEAT1*, confirming that the *NEAT1-PIK3CA/ATM* ceRNA network can mediate α-syn-driven ferroptosis in PD [95].

### lncRNAs in synaptic failure

Synaptic loss is a precursor to neuronal death, leading to cognitive and motor impairments. LncRNAs exhibit significant alterations during the early stages of synaptic impairment and the decline of neuroplasticity in NDDs [96], thus presenting considerable potential as early biomarkers. A study demonstrated that neuronal *BC1* RNA plays a critical role in the protein-synthesis-dependent establishment of neuronal excitation-repression equilibria [97]. Similarly, brain cytoplasmic 200 (*BC200*), a cytoplasm-localized lncRNA predominantly expressed in neurons, can be transported to dendrites and is essential for maintaining long-term synaptic plasticity by targeting translational regulators within the neuronal somatodendritic compartment [98]. The astrocyte-specific lncRNA*PRDM16-DT* was significantly downregulated in postmortem brain tissues from AD patients. Knockdown of lncRNA *PRDM16-DT* impairs astrocytic glutamate uptake and lactate secretion, leading to a reduction in neuronal dendritic spine density in primary mouse astrocytes, while upregulation reverses this dysfunction. These findings indicate that *PRDM16-DT* is involved in regulating synaptic function by modulating astrocytic support for neurons [49]. Additionally, a study investigated the effects of lncRNA metastasis associated lung adenocarcinoma transcript 1 (*MALAT1*) on synapse-related physiological processes, revealing that *MALAT1* was upregulated in various cell types; however, knockout of *MALAT1* in human tumor cells did not significantly affect cell proliferation, the cell cycle, or nuclear architecture. Moreover, genetic loss of *MALAT1* in a knockout mouse model did not result in any apparent phenotype or histological abnormalities, suggesting that the functions of *MALAT1* may be cell-type- or tissue-specific [99].

### lncRNAs linking aging and neurodegeneration

Aging is a significant risk factor for NDDs. Aging-associated NDDs are characterized by progressive neuronal atrophy, neural tissue degeneration, and loss of neurological function, ultimately leading to cognitive and motor impairments. The aging process is further complicated by epigenetic reprogramming, telomere damage, and the senescence-associated secretory phenotype (SASP). The senescence of neural cells exacerbates the incidence of NDDs. Research indicates that regulating the levels of specific lncRNAs in nervous system cells may counteract senescence, representing a promising approach for delaying neurodegeneration (Table 4). Through multifaceted regulatory mechanisms at the transcriptional, post-transcriptional, and epigenetic levels, lncRNAs have emerged as crucial mediators in the progression from organismal aging to neurodegenerative pathogenesis.

**Table 4:** lncRNAs in age-related neurodegeneration

**LncRNA**	**NDDs**	**Changes**	**Function**	**Reference**
NEAT1	/	Up in naturally aged rats	Down-regulation delays cognitive decline in naturally aging rats.	[85]
GAS5	AD	Down in AD patients and aged mouse	Up regulated to inhibit tau phosphorylation and attenuate neuroinflammation.	[101, 102]
SNCA-AS1	PD	Up in PD patients and SH-SY5Y cells	Elicits cellular functions through the regulation of SNCA, with specific modulation of synaptogenesis and senescence.	[103]
PINT	AD/HD	Up in PD and HD patients	Accumulated in several brain regions of AD and HD patients and decreased with healthy brain aging, suggesting a general role in aging and neurodegeneration for this lncRNA.	[104]
TUG1	/	Up in HIV-1 Tat-mediated onset of astrocyte senescence	Up-regulation exacerbates cellular senescence; down-regulation reduces the levels of senescence-associated markers.	[106]

**Abbreviations:** AD, Alzheimer’s disease; GAS5, growth arrest-specific transcript 5; HD, Huntington’s disease; HIV-1, human immunodeficiency virus type 1; lncRNA, long non-coding RNA; NEAT (NEAT1), nuclear paraspeckle assembly transcript 1; PD, Parkinson’s disease; PINT (LINC-PINT), long intergenic non-protein coding RNA, p53 induced transcript; SH-SY5Y, human neuroblastoma cell line; SNCA, synuclein alpha; SNCA-AS1, SNCA antisense RNA 1; TUG1, taurine upregulated 1.

One study explored the mechanisms linking the deficiency of the longevity gene *Klotho* to aging and AD. *Klotho* knockout alters the levels of aging- and cognition-related mRNAs, lncRNAs, microRNAs, and transfer RNA (tRNA) fragments, with 77% of the differentially expressed genes overlapping with the transcriptome of AD mice. Additionally, the *Klotho*knockout mouse model exhibited pathological features consistent with human brain aging and AD. This research suggests that *Klotho*mediates brain aging and neurodegeneration by regulating diverse RNA transcriptomes [100].

Another study indicated that combined cognitive and motor interventions could effectively delay cognitive decline in naturally aging rats by downregulating the expression levels of *NEAT1* in the hippocampus [84].

The lncRNA growth arrest specific 5 (*GAS5*) was found to be downregulated in the brain tissues of AD and aged mice. Its deficiency suppresses neuronal insulin signaling, promotes tau phosphorylation, and exacerbates neuroinflammation. NPC86, a small-molecule compound targeting *GAS5*, stabilizes its expression levels, effectively attenuating neuroinflammation by inhibiting tau phosphorylation [101]. Conversely, *GAS5* was significantly upregulated in the hippocampal tissues of aged mice, suggesting its potential role in driving cellular senescence [102].

The lncRNA synuclein alpha antisense RNA 1 (*SNCA-AS1*), which is the antisense lncRNA of α-synuclein, was implicated in cellular senescence. Overexpression of *SNCA-AS1* upregulates both SNCA and α-synuclein, with alterations in numerous PD-specific genes and pathways associated with aging-related processes [103].

The lncRNA *LINC-PINT* was upregulated in patients with PD and exhibited conspicuous increases in maturing primary culture neurons. Downregulation of *LINC-PINT* exacerbated the death of cultured N2A and SH-SY5Y cells exposed to oxidative stress. Furthermore, *LINC-PINT* accumulation was observed in various brain regions of Alzheimer’s and HD patients, decreasing with healthy brain aging [104].

The lncRNA *BC089918* was significantly downregulated following sciatic nerve injury and promotes neurite outgrowth in dorsal root ganglion neurons, suggesting its relevance to nerve injury and regenerative repair in the elderly [105].

The lncRNA *TUG1* was upregulated in primary human astrocytes induced to undergo cellular senescence by exposure to the human immunodeficiency virus (HIV) trans-activator of transcription. Downregulation of *TUG1* expression reduced the levels of senescence-associated markers [106].

## Clinical applications

LncRNAs are implicated in multiple biological processes and are closely associated with the onset and progression of NDDs. They can be released into various body fluids, including peripheral blood (serum and plasma), cerebrospinal fluid (CSF), and saliva, through active secretion or apoptotic pathways. Studies have demonstrated that detecting lncRNAs in the body fluids of patients with NDDs is an effective diagnostic approach.

### lncRNAs as liquid biopsy biomarkers

In a study involving 88 patients with AD and 72 healthy controls, plasma expression levels of lncRNA *17A*,*51A*, *BACE1*, and *BC200* were found to be upregulated. Plasma *BACE1* levels were significantly elevated in AD patients compared to healthy controls. Receiver operating characteristic curve analysis revealed an area under the curve (AUC) of 0.667 for *BACE1-AS* [107]. Furthermore, a separate study reported that plasma *BACE1* levels were significantly regulated in AD patients, distinguishing them from healthy controls with 88% specificity for AD [108]. These observations suggest that *BACE1* may serve as a potential biomarker for AD.

The lncRNA *NEAT1* was significantly upregulated in the CSF and peripheral blood of PD patients. Downregulation of *NEAT1* reduced cell apoptosis in a PD model using PC12 cells treated with MPP^+^. Additionally, knockdown of *NEAT1* decreased inflammatory infiltration in brain tissue and cell apoptosis in the PD mouse model, indicating that *NEAT1* is a potential biomarker for PD and a target for therapy [109].

A bioinformatics analysis of AD-related databases identified lncRNA *RN7SK* as a key gene involved in AD pathogenesis and associated with inflammatory responses. Notably, *RN7SK* was significantly upregulated in peripheral blood mononuclear cell (PBMC) samples from AD patients (*P* ═ 0.006) [110].

A comprehensive bioinformatics analysis of datasets from the GEO, which included 589 samples from patients with AD and healthy controls, revealed that a combined biomarker panel consisting of five lncRNAs—*SNHG14*, prostate androgen-regulated transcript 1 (*PART1*), nicotinamide nucleotide transhydrogenase antisense RNA 1 (*NNT-AS1*), *AC093010.3*, and *ARMCX5-GPRASP2*—demonstrated strong diagnostic efficacy for AD. Notably, *PART1* was significantly downregulated, whereas *SNHG14* was significantly upregulated in the serum samples of AD patients, revealing the potential roles of lncRNAs in the pathogenesis of AD [111].

A meta-analysis involving datasets from 553 patients with AD and 513 healthy controls identified significant upregulation of *BACE1-AS*, *NEAT1*, and *GAS5* in plasma samples from AD patients. The pooled sensitivity, specificity, and AUC of lncRNAs for AD identification were reported as 0.74 (95% CI [0.63, 0.82], I^2^ ═ 79.2%), 0.88 (95% CI [0.75, 0.94], I^2^ ═ 88.9%), and 0.86 (95% CI [0.82, 0.88]), respectively. This study illustrates the high diagnostic accuracy of lncRNAs for AD, highlighting their potential as liquid biopsy biomarkers for the disease [112].

Fifteen epigenetically regulated lncRNAs were selected to develop a blood-based diagnostic model and nomogram for AD. The receiver operating characteristic (ROC) curve, along with decision and calibration curves, indicated strong predictive performance of the model. Furthermore, the methylation levels of lncRNAs in blood samples may serve as indicators for diagnosing AD, providing new insights for the development of non-invasive liquid biopsy markers for the disease [113].

Similar investigations have been conducted in patients with PD. A study utilizing Gene Ontology (GO) and Kyoto Encyclopedia of Genes and Genomes (KEGG) enrichment analyses of peripheral blood plasma from six PD patients and six healthy controls revealed that *lncMTG2-1:1*, *lnc-CTSD-5:1*, *lnc-PCCA-3:1*, *lnc-VTCN1-3:1*, *lnc-ZNF25-7:1*, and*lnc-DAZ3-1:1* may represent key lncRNAs in PD. Notably, *lnc-CTSD-5:1* exhibited the most significant alterations, and silencing this lncRNA restored tyrosine expression levels in MPP^+^-treated SH-SY5Y cells [114].

Various cell types in the central nervous system (CNS), including neurons, astrocytes, oligodendrocytes, microglia, and embryonic neural stem cells, are capable of secreting exosomes. Research has demonstrated that lncRNAs can be selectively packaged into these exosomes, which can traverse the blood-brain barrier and transport brain-derived RNAs, proteins, and lipids into peripheral blood, CSF, saliva, and other bodily fluids. Consequently, exosomal lncRNAs can accurately reflect pathological changes in the CNS and significantly enhance diagnostic specificity, enabling non-invasive or minimally invasive disease detection. They represent a promising direction for future liquid biopsy applications.

The lncRNA *BACE1-AS* is notably upregulated in exosomes isolated from the plasma of AD patients [107]. Additionally, lncRNAs prostate cancer antigen 3 (*PCA3*) and *RP11-462G22.1* are significantly upregulated in extracellular vesicles (EVs) isolated from the CSF of AD patients [115].

A study involving PD patients and age- and sex-matched healthy controls evaluated lncRNA levels extracted from exosomes in plasma samples, identifying 15 upregulated and 24 downregulated exosomal lncRNAs in the PD cohort. Bioinformatics analyses revealed that *lnc-MKRN2-42:1* was positively correlated with Movement Disorder Society-Unified Parkinson’s Disease Rating Scale (MDS-UPDRS) III scores, highlighting significant differences in exosomal lncRNA profiles between PD patients and controls [116].

To date, few studies have examined EV-derived lncRNAs directly associated with AD. Furthermore, lncRNAs carried by exosomes from diverse cellular origins and varying particle sizes demonstrate notable differences in types, expression levels, and regulatory roles in disease, indicating that current research in this area remains insufficient. Continued investigation is warranted to explore these avenues further.

### lncRNAs as therapeutic targets

Therapeutic strategies targeting lncRNAs are classified into two main categories: the first involves inhibiting the expression or function of pathogenic lncRNAs, while the second focuses on restoring or enhancing the expression levels of protective lncRNAs. Knockdown of *SNHG1* reversed cell apoptosis, mitochondrial membrane potential (MMP) alterations, caspase-3 activity, and the expression levels of apoptosis-related proteins in AD cell models induced by Aβ25-35[117]. Additionally, studies indicated that Wilms tumor 1 antisense RNA (*WT1-AS*) is downregulated in Aβ25-35-treated SH-SY5Y cells; however, overexpression of *WT1-AS* effectively inhibited cell apoptosis and improved learning and memory abilities in AD mouse models [118].

Antisense oligonucleotides (ASOs) present a promising therapeutic approach. ASOs can modulate gene expression at the post-transcriptional level by targeting specific RNAs, particularly those that are challenging to regulate using small molecules or antibody drugs. Research has shown that multiple ASO-targeting lncRNAs significantly improved pathological conditions and behavior in mouse models, with some progressing to early clinical trials demonstrating safety and tolerability [119]. Recent decades have seen the Journal of Clinical Investigation (JCI) publish several significant preclinical advances, underscoring the clinical translational potential of ASOs. Presently, two ASOs have gained approval for treating degenerative motor neuron diseases, such as spinal muscular atrophy (SMA) and ALS [120]. BIIB080 was the first ASO to enter clinical trials targeting tau expression for mild AD [121].

BIIB067, an ASO targeting SOD1, effectively downregulated SOD1 mRNA expression levels by binding to specific regions of the SOD1 pre-mRNA. Administration of this ASO into the CSF of mice, rats, and monkeys expressing human SOD1G93A significantly reduced SOD1 mRNA levels in the brain and spinal cord, thereby delaying disease onset and prolonging survival in ALS animal models [122].

Treatment with Jacifusen or ION363, targeting FUS with an ASO, could delay motor neuron degeneration in ALS mouse models by reducing FUS protein levels in the brain and spinal cord [123].

*TUG1*-targeting ASO (*TUG1*-ASO) represents a specific nucleic acid-based therapeutic that incorporates a drug delivery system, demonstrating antitumor efficacy against glioblastoma (GB) in mouse models. The study enrolled patients aged 18–75 years with recurrent glioblastoma (rGB) following postoperative temozolomide combined with radiotherapy to evaluate the safety, tolerability, and maximum tolerated dose (MTD) of *TUG1*-ASO [124]. While this research primarily addresses malignant glioma rather than traditional NDDs, it provides critical evidence for the therapeutic potential of lncRNAs in neurodegenerative disorders. Given their stability and feasibility for brain delivery, ASOs are emerging as a highly promising therapeutic strategy, with numerous ASOs currently in preclinical and clinical development. There is hope for the widespread application of these therapeutic technologies in treating NDDs.

The clustered regularly interspaced short palindromic repeats / CRISPR-associated protein 9 (CRISPR/Cas9) system has introduced novel therapeutic approaches for lncRNAs in recent years. Knocking out pathogenic lncRNAs via the CRISPR/Cas9 system or specifically upregulating protective lncRNAs through CRISPR activation (CRISPRa) offers promising new strategies for intervening in the pathological progression of NDDs [125]. In HD and various nucleotide repeat expansion disorders, CRISPR/Cas9 genome editing has successfully been employed to eliminate pathogenic repeat sequences or gene fragments [126]. While much research has focused on protein-coding sequences, this paradigm can be extended to lncRNAs by disrupting lncRNA promoters, splice sites, or functional domains, thereby regulating their expression levels in neuronal cells. We believe the CRISPR/Cas9 system can also effectively regulate lncRNA expression in neurons through such mechanisms.

Exosome membrane-camouflaged nanocarriers have emerged as a novel strategy for targeted drug delivery in NDDs [127]. A study isolating exosomes from human umbilical cord mesenchymal stem cells (hUC-MSCs) (WT-Exo) and employing small interfering RNA (siRNA) to establish a knockdown of *lncRNA9969* (KD-Exo) revealed that WT-Exo significantly reduced cellular damage and improved Aβ clearance in Aβ-injured SH-SY5Y cells. In contrast, the protective effect of KD-Exo was notably diminished. In *APP/PS1* mice, treatment with WT-Exo significantly improved spatial memory deficits and upregulated hippocampal expression of synaptic proteins such as synaptophysin (Syn) and brain-derived neurotrophic factor (BDNF). This study indicates that hUC-MSC-derived exosomes exert neuroprotective effects by delivering functional *lncRNA9969*, elucidating the central role of exosomal lncRNAs in AD.

## Challenges and prospects

LncRNAs have emerged as critical factors in NDDs due to their essential roles in gene expression regulation, neuroinflammation, and neuronal survival [128]. However, the complex pathological mechanisms underlying NDDs, the intricate pathological networks involving multisystem interactions, and the challenges posed by the blood-brain barrier significantly hinder therapeutic strategies targeting lncRNAs [129].

From a molecular mechanism perspective, despite the known involvement of lncRNAs in oxidative stress, neuroinflammation, and protein homeostasis within core pathological processes, several obstacles remain. The human genome harbors a vast array of annotated lncRNAs, yet most of their functions remain poorly understood, limiting comprehensive assessments of their biological effects in NDDs. Furthermore, lncRNAs exhibit tissue and cell type specificity, making it challenging to obtain high-quality human brain tissue samples for dynamic functional studies.

In biomarker development, lncRNAs are recognized as promising invasive and high-sensitivity biomarkers due to their stability and tissue specificity in CSF and peripheral blood. However, the field encounters difficulties in validating specificity and translating these findings into clinical practice. To enhance diagnostic accuracy, lncRNA detection can be integrated with existing technologies. For instance, protein amplification techniques can currently detect ultra-low concentrations of abnormal proteins in biological fluids, facilitating the creation of multi-dimensional early diagnostic models for more precise disease assessment [130]. The application of artificial intelligence and multimodal sensing technologies will enable highly sensitive detection and real-time monitoring of lncRNA biomarkers, laying the groundwork for early disease diagnosis in the future. Additionally, cutting-edge technologies such as single-cell transcriptome sequencing and spatial transcriptomics may pave the way for early diagnosis of NDDs.

Regarding therapeutic targets, dysregulated lncRNA expression during NDDs presents an opportunity to correct imbalanced gene expression networks by targeting key lncRNA molecules, constituting a vital direction for exploring novel therapeutic strategies in NDDs. Nevertheless, the application of ASOs in NDDs faces significant challenges, primarily due to the blood-brain barrier. As negatively charged biomacromolecules, ASOs struggle to penetrate the blood-brain barrier effectively and are prone to clearance from the central nervous system [131]. Future efforts must focus on enhancing the efficiency and safety of drug delivery across the blood-brain barrier, optimizing the targeted delivery of lncRNA-based therapeutics through nanoplatforms and biomaterials. Moreover, while the CRISPR/Cas9 system enables the modification of pathogenic lncRNAs, off-target effects and safety concerns pose challenges to clinical application.

LncRNAs hold substantial potential for application in NDDs, yet clinical translation faces numerous challenges. Despite these hurdles, the ongoing development of high-throughput screening technologies, bioinformatics algorithms, and novel drug delivery systems, combined with the integration of molecular neurobiology, nanotechnology, artificial intelligence, and precision medicine, is anticipated to provide new directions for NDD treatment and clinical drug development.

## Conclusion

Given their high expression specificity, multilayered regulatory modes, and broad pathological relevance, lncRNAs have emerged as a focal point in neurodegenerative disease research. They serve as key regulators mediating pathological events such as abnormal protein aggregation, neuroinflammation, mitochondrial dysfunction, synaptic damage, and aberrant cell death, while also representing ideal molecular targets for early diagnosis, monitoring, and targeted therapies for these disorders. With ongoing advancements in innovative fields such as biomacromolecular phase separation, ferroptosis, exosomes, and spatial transcriptomics, research targeting lncRNAs is rapidly transitioning from elucidating basic mechanisms to achieving clinical translation. In the future, the establishment of a precision diagnosis and treatment system centered on lncRNAs may yield novel intervention strategies for the prevention and treatment of NDDs.

## References

[1] Dugger BN, Dickson DW (2017). Pathology of neurodegenerative diseases. Cold Spring Harb Perspect Biol.

[2] Schepici G, Bramanti P, Mazzon E (2020). Efficacy of sulforaphane in neurodegenerative diseases. Int J Mol Sci.

[3] Hardy J (2001). The genetic causes of neurodegenerative diseases. J Alzheimers Dis.

[4] Chen S, Liu M, Yi W, Li H, Yu Q (2025). Micropeptides derived from long non-coding RNAs: computational analysis and functional roles in breast cancer and other diseases. Gene.

[5] Cajigas I, Chakraborty A, Swyter KR, Luo H, Bastidas M, Nigro M (2018). The Evf2 ultraconserved enhancer lncRNA functionally and spatially organizes megabase distant genes in the developing forebrain. Mol Cell.

[6] Huang J, Jiang B, Li GW, Zheng D, Li M, Xie X (2022). m(6)A-modified lincRNA Dubr is required for neuronal development by stabilizing YTHDF1/3 and facilitating mRNA translation. Cell Rep.

[7] Bernard D, Prasanth KV, Tripathi V, Colasse S, Nakamura T, Xuan Z (2010). A long nuclear-retained non-coding RNA regulates synaptogenesis by modulating gene expression. EMBO J.

[8] Han CL, Ge M, Liu YP, Zhao XM, Wang KL, Chen N (2018). LncRNA H19 contributes to hippocampal glial cell activation via JAK/STAT signaling in a rat model of temporal lobe epilepsy. J Neuroinflammation.

[9] Stein CS, Jadiya P, Zhang X, McLendon JM, Abouassaly GM, Witmer NH (2018). Mitoregulin: a lncRNA-encoded microprotein that supports mitochondrial supercomplexes and respiratory efficiency. Cell Rep.

[10] Bartolomei MS, Zemel S, Tilghman SM (1991). Parental imprinting of the mouse H19 gene. Nature.

[11] Okazaki Y, Furuno M, Kasukawa T, Adachi J, Bono H, Kondo S (2002). Analysis of the mouse transcriptome based on functional annotation of 60,770 full-length cDNAs. Nature.

[12] Amaral P, Carbonell-Sala S, De La Vega FM, Faial T, Frankish A, Gingeras T (2023). The status of the human gene catalogue. Nature.

[13] Fang S, Zhang L, Guo J, Niu Y, Wu Y, Li H (2018). NONCODEV5:a comprehensive annotation database for long non-coding RNAs. Nucleic Acids Res.

[14] Lander ES, Linton LM, Birren B, Nusbaum C, Zody MC, Baldwin J (2001). Initial sequencing and analysis of the human genome. Nature.

[15] Rinn JL, Chang HY (2012). Genome regulation by long noncoding RNAs. Annu Rev Biochem.

[16] Quinn JJ, Chang HY (2016). Unique features of long non-coding RNA biogenesis and function. Nat Rev Genet.

[17] Ørom UA, Derrien T, Beringer M, Gumireddy K, Gardini A, Bussotti G (2010). Long noncoding RNAs with enhancer-like function in human cells. Cell.

[18] Engreitz JM, Ollikainen N, Guttman M (2016). Long non-coding RNAs: spatial amplifiers that control nuclear structure and gene expression. Nat Rev Mol Cell Biol.

[19] Engreitz JM, Haines JE, Perez EM, Munson G, Chen J, Kane M (2016). Local regulation of gene expression by lncRNA promoters, transcription and splicing. Nature.

[20] Statello L, Guo CJ, Chen LL, Huarte M (2021). Gene regulation by long non-coding RNAs and its biological functions. Nat Rev Mol Cell Biol.

[21] Wang KC, Chang HY (2011). Molecular mechanisms of long noncoding RNAs. Mol Cell.

[22] Tsai MC, Manor O, Wan Y, Mosammaparast N, Wang JK, Lan F (2010). Long noncoding RNA as modular scaffold of histone modification complexes. Science.

[23] Button AC, Hall SD, Ashley EL, McHugh CA (2024). Dissection of protein and RNA regions required for SPEN binding to XIST A-repeat RNA. RNA.

[24] Nagano T, Mitchell JA, Sanz LA, Pauler FM, Ferguson-Smith AC, Feil R (2008). The Air noncoding RNA epigenetically silences transcription by targeting G9a to chromatin. Science.

[25] Huarte M, Guttman M, Feldser D, Garber M, Koziol MJ, Kenzelmann-Broz D (2010). A large intergenic noncoding RNA induced by p53 mediates global gene repression in the p53 response. Cell.

[26] Martianov I, Ramadass A, Serra Barros A, Chow N, Akoulitchev A (2007). Repression of the human dihydrofolate reductase gene by a non-coding interfering transcript. Nature.

[27] Salmena L, Poliseno L, Tay Y, Kats L, Pandolfi PP (2011). A ceRNA hypothesis: the Rosetta Stone of a hidden RNA language?. Cell.

[28] Tripathi V, Ellis JD, Shen Z, Song DY, Pan Q, Watt AT (2010). The nuclear-retained noncoding RNA MALAT1 regulates alternative splicing by modulating SR splicing factor phosphorylation. Mol Cell.

[29] Yoon JH, Abdelmohsen K, Srikantan S, Yang X, Martindale JL, De S (2012). LincRNA-p21 suppresses target mRNA translation. Mol Cell.

[30] Zhao Y, Chai X, Peng J, Zhu Y, Dong R, He J (2025). Proline exacerbates hepatic gluconeogenesis via paraspeckle-dependent mRNA retention. Nat Metab.

[31] Carrieri C, Cimatti L, Biagioli M, Beugnet A, Zucchelli S, Fedele S (2012). Long non-coding antisense RNA controls Uchl1 translation through an embedded SINEB2 repeat. Nature.

[32] Zhang X, Yuan L, Zhang W, Zhang Y, Wu Q, Li C (2024). Liquid–liquid phase separation in diseases. MedComm (2020).

[33] Su X, Ditlev JA, Hui E, Xing W, Banjade S, Okrut J (2016). Phase separation of signaling molecules promotes T cell receptor signal transduction. Science.

[34] Bayona-Hernandez A, Guerra S, Jiménez-Ramirez IA, Sztacho M, Hozak P, Rodriguez-Zapata LC (2023). LIPRNAseq: a method to discover lipid interacting RNAs by sequencing. Mol Biol Rep.

[35] Yao Y, Yan C, Huang H, Wang S, Li J, Chen Y (2025). LncRNA-MEG3 regulates muscle mass and metabolic homeostasis by facilitating SUZ12 liquid–liquid phase separation. Adv Sci (Weinh).

[36] Zhang N, Wen K (2024). The role of lncRNA binding to RNA-binding proteins to regulate mRNA stability in cancer progression and drug resistance mechanisms (Review). Oncol Rep.

[37] Renganathan A, Minaya MA, Broder M, Alfradique-Dunham I, Miller RL, Dhavale DD (2025). A novel lncRNA FAM151B-DT regulates degradation of aggregation prone proteins. Mol Psychiatry.

[38] Prashad S, Gopal PP (2021). RNA-binding proteins in neurological development and disease. RNA Biol.

[39] Tao Y, Zhang Q, Wang H, Yang X, Mu H (2024). Alternative splicing and related RNA binding proteins in human health and disease. Signal Transduct Target Ther.

[40] Hashemi M, Shafiei Asheghabadi P, Moassesfar M, Mashhadikhan S, Nasirzade S, Vasheghani Farahani A (2026). MicroRNAs and long non-coding RNAs affect the mechanisms involved in age-related neurodegeneration in a manner depending on RNA-binding proteins. Mol Neurobiol.

[41] Jiang W, Peng A, Chen Y, Pang B, Zhang Z (2020). Long non-coding RNA EBLN3P promotes the recovery of the function of impaired spiral ganglion neurons by competitively binding to miR-204-5p and regulating TMPRSS3 expression. Int J Mol Med.

[42] Choi SW, Kim HW, Nam JW (2019). The small peptide world in long noncoding RNAs. Brief Bioinform.

[43] Wen T, Liang D, Wang T, Hu W, Li Y, Shi L (2026). LncRNA-encoded micropeptides: emerging regulators of cancer metabolism and precision oncology. Curr Opin Cell Biol.

[44] Kong J, Su X, Zhou C, Lin W, Lin A, Tang J (2025). Micropeptides encoded by noncoding RNAs: biological functions and roles in diseases. Research (Wash D C).

[45] Yi Q, Feng J, Lan W, Shi H, Sun W, Sun W (2024). CircRNA and lncRNA-encoded peptide in diseases, an update review. Mol Cancer.

[46] Zheng X, Wang M, Liu S, Chen H, Li Y, Yuan F (2023). A lncRNA-encoded mitochondrial micropeptide exacerbates microglia-mediated neuroinflammation in retinal ischemia/reperfusion injury. Cell Death Dis.

[47] Lisi M, Santini T, D’Andrea T, Salvatori B, Setti A, Paiardini A (2025). SERTM2:a neuroactive player in the world of micropeptides. EMBO Rep.

[48] Li MM, Shi MJ, Feng CC, Yu ZY, Bai XF, Lu L (2024). LncRNA KCNQ1OT1 promotes NLRP3 inflammasome activation in Parkinson’s disease by regulating pri-miR-186/mature miR-186/NLRP3 axis. Biochim Biophys Acta Mol Basis Dis.

[49] Schröder S, Fuchs U, Gisa V, Pena T, Krüger DM, Hempel N (2024). PRDM16-DT is a novel lncRNA that regulates astrocyte function in Alzheimer’s disease. Acta Neuropathol.

[50] Schröder S, Fuchs U, Gisa V, Pena T, Krüger DM, Hempel N PRDM16-DT: a brain and astrocyte-specific lncRNA implicated in Alzheimer’s disease.

[51] Khan MR, Avino M, Wellinger RJ, Laurent B (2023). Distinct regulatory functions and biological roles of lncRNA splice variants. Mol Ther Nucleic Acids.

[52] Schröder S, Sakib MS, Krüger DM, Pena T, Burkhardt S, Schütz AL (2025). LncRNA 3222401L13Rik is upregulated in aging astrocytes and regulates neuronal support function through interaction with Npas3. Noncoding RNA.

[53] Trushina E, McMurray CT (2007). Oxidative stress and mitochondrial dysfunction in neurodegenerative diseases. Neuroscience.

[54] Kumar J, Mohammad G, Alka K, Kowluru RA (2023). Mitochondrial genome-encoded long noncoding RNA and mitochondrial stability in diabetic retinopathy. Diabetes.

[55] Kumar J, Kowluru RA (2024). Mitochondrial DNA transcription and mitochondrial genome-encoded long noncoding RNA in diabetic retinopathy. Mitochondrion.

[56] Xiong W, Lu L, Li J (2024). Long non-coding RNAs with essential roles in neurodegenerative disorders. Neural Regen Res.

[57] Kowluru RA, Kumar J (2025). Mitochondrial fragmentation and long noncoding RNA MALAT1 in diabetic retinopathy. Int J Mol Sci.

[58] Xiong W, Xu K, Sun JK, Liu S, Zhao B, Shi J (2024). The mitochondrial long non-coding RNA lncMtloop regulates mitochondrial transcription and suppresses Alzheimer’s disease. EMBO J.

[59] Olazagoitia-Garmendia A, Rojas-Márquez H, Trobisch T, Moreno-Castro C, Rodriguez Etxebarria A, Mentxaka J (2025). An inflammation-associated lncRNA induces neuronal damage via mitochondrial dysfunction. Mol Ther Nucleic Acids.

[60] Katopodi V, Marino A, Pateraki N, Verheyden Y, Cinque S, Jimenez EL (2025). The long non-coding RNA ROSALIND protects the mitochondrial translational machinery from oxidative damage. Cell Death Differ.

[61] Lei G, Zhuang L, Gan B (2022). Targeting ferroptosis as a vulnerability in cancer. Nat Rev Cancer.

[62] Cai Y, Liu J, Wang B, Sun M, Yang H (2022). Microglia in the neuroinflammatory pathogenesis of Alzheimer’s disease and related therapeutic targets. Front Immunol.

[63] Lin P, Wang J, Li Y, Li G, Wang Y (2024). LINC00472 regulates ferroptosis of neurons in Alzheimer’s disease via FOXO1. Dement Geriatr Cogn Disord.

[64] Yin X, Qiu Y, Zhao C, Zhou Z, Bao J, Qian W (2021). The role of amyloid-beta and tau in the early pathogenesis of Alzheimer’s disease. Med Sci Monit.

[65] Faghihi MA, Modarresi F, Khalil AM, Wood DE, Sahagan BG, Morgan TE (2008). Expression of a noncoding RNA is elevated in Alzheimer’s disease and drives rapid feed-forward regulation of beta-secretase. Nat Med.

[66] Liu T, Huang Y, Chen J, Chi H, Yu Z, Wang J (2014). Attenuated ability of BACE1 to cleave the amyloid precursor protein via silencing long noncoding RNA BACE1-AS expression. Mol Med Rep.

[67] Yi J, Chen B, Yao X, Lei Y, Ou F, Huang F (2019). Upregulation of the lncRNA MEG3 improves cognitive impairment, alleviates neuronal damage, and inhibits activation of astrocytes in hippocampus tissues in Alzheimer’s disease through inactivating the PI3K/Akt signaling pathway. J Cell Biochem.

[68] Massone S, Vassallo I, Fiorino G, Castelnuovo M, Barbieri F, Borghi R (2011). 17A, a novel non-coding RNA, regulates GABA B alternative splicing and signaling in response to inflammatory stimuli and in Alzheimer disease. Neurobiol Dis.

[69] Gu C, Chen C, Wu R, Dong T, Hu X, Yao Y (2018). Long noncoding RNA EBF3-AS promotes neuron apoptosis in Alzheimer’s disease. DNA Cell Biol.

[70] Zhang T, Pang P, Fang Z, Guo Y, Li H, Li X (2018). Expression of BC1 impairs spatial learning and memory in Alzheimer’s disease via APP translation. Mol Neurobiol.

[71] Ke S, Yang Z, Yang F, Wang X, Tan J, Liao B (2019). Long noncoding RNA NEAT1 aggravates Aβ-induced neuronal damage by targeting miR-107 in Alzheimer’s disease. Yonsei Med J.

[72] Scheele C, Nielsen AR, Walden TB, Sewell DA, Fischer CP, Brogan RJ (2007). Altered regulation of the PINK1 locus: a link between type 2 diabetes and neurodegeneration?. FASEB J.

[73] Yan W, Chen ZY, Chen JQ, Chen HM (2018). LncRNA NEAT1 promotes autophagy in MPTP-induced Parkinson’s disease through stabilizing PINK1 protein. Biochem Biophys Res Commun.

[74] Sun Q, Zhang Y, Wang S, Yang F, Cai H, Xing Y (2021). NEAT1 decreasing suppresses Parkinson’s disease progression via acting as miR-1301-3p sponge. J Mol Neurosci.

[75] Lu M, Sun WL, Shen J, Wei M, Chen B, Qi YJ (2018). LncRNA-UCA1 promotes PD development by upregulating SNCA. Eur Rev Med Pharmacol Sci.

[76] Li Y, Fang J, Zhou Z, Zhou Q, Sun S, Jin Z (2020). Downregulation of lncRNA BACE1-AS improves dopamine-dependent oxidative stress in rats with Parkinson’s disease by upregulating microRNA-34b-5p and downregulating BACE1. Cell Cycle.

[77] Wang N, Wang M, You Y, An J, Jia R, Zhao B (2026). Lnc-USP28-6/ZBTB16 axis orchestrates NLRP3 inflammasome activation and α-synuclein SUMOylation to drive Parkinson’s disease pathogenesis. Brain Behav Immun.

[78] Nishimoto Y, Nakagawa S, Hirose T, Okano HJ, Takao M, Shibata S (2013). The long non-coding RNA nuclear-enriched abundant transcript 1_2 induces paraspeckle formation in the motor neuron during the early phase of amyotrophic lateral sclerosis. Mol Brain.

[79] Chung DW, Rudnicki DD, Yu L, Margolis RL (2011). A natural antisense transcript at the Huntington’s disease repeat locus regulates HTT expression. Hum Mol Genet.

[80] Yang R, Yang B, Liu W, Tan C, Chen H, Wang X (2023). Emerging role of non-coding RNAs in neuroinflammation mediated by microglia and astrocytes. J Neuroinflammation.

[81] Xie J, Lan Y, Zou C, He J, Huang Q, Zeng J (2024). Single-nucleus analysis reveals microenvironment-specific neuron and glial cell enrichment in Alzheimer’s disease. BMC Genomics.

[82] Ma P, Li Y, Zhang W, Fang F, Sun J, Liu M (2019). Long non-coding RNA MALAT1 inhibits neuron apoptosis and neuroinflammation while stimulates neurite outgrowth and its correlation with miR-125b mediates PTGS2, CDK5 and FOXQ1 in Alzheimer’s disease. Curr Alzheimer Res.

[83] Duan R, Wang SY, Wei B, Deng Y, Fu XX, Gong PY (2021). Angiotensin-(1–7) analogue AVE0991 modulates astrocyte-mediated neuroinflammation via lncRNA SNHG14/miR-223-3p/NLRP3 pathway and offers neuroprotection in a transgenic mouse model of Alzheimer’s disease. J Inflamm Res.

[84] Li T, Tao X, Sun R, Han C, Li X, Zhu Z (2023). Cognitive-exercise dual-task intervention ameliorates cognitive decline in natural aging rats via inhibiting the promotion of lncRNA NEAT1/miR-124-3p on caveolin-1-PI3K/Akt/GSK3β pathway. Brain Res Bull.

[85] Liu Y, Feng D, Liu F, Liu Y, Zuo F, Wang Y (2024). LncRNA MALAT1 facilitates Parkinson’s disease progression by increasing SOCS3 promoter methylation. Gerontology.

[86] Feng D, Liu Y, Zuo F, Liu F, Liu Y, Wang Y (2024). LncRNA SOX21-AS1 promotes activation of BV2 cells via epigenetical silencing of SOCS3 and aggravates Parkinson’s disease. Gerontology.

[87] Xiao X, Tan Z, Jia M, Zhou X, Wu K, Ding Y (2021). Long noncoding RNA SNHG1 knockdown ameliorates apoptosis, oxidative stress and inflammation in models of Parkinson’s disease by inhibiting the miR-125b-5p/MAPK1 axis. Neuropsychiatr Dis Treat.

[88] Benarroch E (2023). What is the role of ferroptosis in neurodegeneration?. Neurology.

[89] Zhou M, Xu K, Ge J, Luo X, Wu M, Wang N (2024). Targeting ferroptosis in Parkinson’s disease: mechanisms and emerging therapeutic strategies. Int J Mol Sci.

[90] Tan Y, Tang W, Xiao W, Huang R, Li X, Peng W (2023). LncRNA-associated ceRNA network revealing the potential regulatory roles of ferroptosis and immune infiltration in Alzheimer’s disease. Front Aging Neurosci.

[91] He YJ, Cong L, Liang SL, Ma X, Tian JN, Li H (2022). Discovery and validation of ferroptosis-related molecular patterns and immune characteristics in Alzheimer’s disease. Front Aging Neurosci.

[92] Gu X, Song Y, Liu X, Cheng Z, Min J, Zhang Y (2024). METTL14-mediated m6A modification of TUG1 represses ferroptosis in Alzheimer’s disease via inhibiting GDF15 ubiquitination. Front Biosci (Landmark Ed).

[93] Zhao J, Wan XN, Zhu JP, Liu QC, Gan L (2022). LncRNA NEAT1 promoted MPP+-induced ferroptosis via regulating miR-150-5p/BAP1 pathway in SK-N-SH cells. Acta Neurobiol Exp (Wars).

[94] Li T, Guo J (2024). Identifying the NEAT1/miR-26b-5p/S100A2 axis as a regulator in Parkinson’s disease based on the ferroptosis-related genes. PLoS One.

[95] Wang M, Li T, Gao R, Zhang Y, Han Y (2023). Identifying the potential genes in alpha synuclein driving ferroptosis of Parkinson’s disease. Sci Rep.

[96] Chanda K, Grinman E, Clark K, Sadhu A, Raveendra B, Swarnkar S (2025). The lncRNA Gas5 is an activity-responsive scaffold that mediates cAMP-dependent synaptic plasticity. Sci Signal.

[97] Zhong J, Chuang SC, Bianchi R, Zhao W, Lee H, Fenton AA (2009). BC1 regulation of metabotropic glutamate receptor-mediated neuronal excitability. J Neurosci.

[98] Mus E, Hof PR, Tiedge H (2007). Dendritic BC200 RNA in aging and in Alzheimer’s disease. Proc Natl Acad Sci U S A.

[99] Eißmann M, Gutschner T, Hämmerle M, Günther S, Caudron-Herger M, Groß M (2012). Loss of the abundant nuclear non-coding RNA MALAT1 is compatible with life and development. RNA Biol.

[100] Dubnov S, Bennett ER, Yayon N, Yakov O, Bennett DA, Seshadri S (2024). Knockout of the longevity gene Klotho perturbs aging and Alzheimer’s disease-linked brain microRNAs and tRNA fragments. Commun Biol.

[101] Patel RS, Lui A, Hudson C, Moss L, Sparks RP, Hill SE (2023). Small molecule targeting long noncoding RNA GAS5 administered intranasally improves neuronal insulin signaling and decreases neuroinflammation in an aged mouse model. Sci Rep.

[102] Meier I, Fellini L, Jakovcevski M, Schachner M, Morellini F (2010). Expression of the snoRNA host gene gas5 in the hippocampus is upregulated by age and psychogenic stress and correlates with reduced novelty-induced behavior in C57BL/6 mice. Hippocampus.

[103] Rey F, Pandini C, Messa L, Launi R, Barzaghini B, Zangaglia R (2021). α-Synuclein antisense transcript SNCA-AS1 regulates synapses- and aging-related genes suggesting its implication in Parkinson’s disease. Aging Cell.

[104] Simchovitz A, Hanan M, Yayon N, Lee S, Bennett ER, Greenberg DS (2020). A lncRNA survey finds increases in neuroprotective LINC-PINT in Parkinson’s disease substantia nigra. Aging Cell.

[105] Yu B, Zhou S, Hu W, Qian T, Gao R, Ding G (2013). Altered long noncoding RNA expressions in dorsal root ganglion after rat sciatic nerve injury. Neurosci Lett.

[106] Pillai PP, Kannan M, Sil S, Singh S, Thangaraj A, Chivero ET (2023). Involvement of lncRNA TUG1 in HIV-1 Tat-induced astrocyte senescence. Int J Mol Sci.

[107] Fotuhi SN, Khalaj-Kondori M, Hoseinpour Feizi MA, Talebi M (2019). Long non-coding RNA BACE1-AS may serve as an Alzheimer’s disease blood-based biomarker. J Mol Neurosci.

[108] Feng L, Liao YT, He JC, Xie CL, Chen SY, Fan HH (2018). Plasma long non-coding RNA BACE1 as a novel biomarker for diagnosis of Alzheimer disease. BMC Neurol.

[109] Wang Y, Li Z, Li J, Sun C (2024). LncRNA NEAT1 promotes MPP+-induced injury of PC12 cells and accelerates the progression of Parkinson’s disease in mice through FUS-mediated inhibition of PI3K/AKT/mTOR signalling pathway. Exp Gerontol.

[110] Kazemi M, Naghdi Sadeh R, Shekari Khaniani M, Rezazadeh M, Derakhshan SM, Ghafouri-Fard S (2024). Identification of RN7SK lncRNA as a novel biomarker in Alzheimer’s disease using bioinformatics and expression analysis. Sci Rep.

[111] Huaying C, Xing J, Luya J, Linhui N, Di S, Xianjun D (2020). A signature of five long non-coding RNAs for predicting the prognosis of Alzheimer’s disease based on competing endogenous RNA networks. Front Aging Neurosci.

[112] Shobeiri P, Alilou S, Jaberinezhad M, Zare F, Karimi N, Maleki S (2023). Circulating long non-coding RNAs as novel diagnostic biomarkers for Alzheimer’s disease (AD): a systematic review and meta-analysis. PLoS One.

[113] Tian G, Zheng D, Zhang N, Deng W, Xu J, Huang C (2025). Association of blood-based DNA methylation of lncRNAs with Alzheimer’s disease diagnosis. Clin Epigenetics.

[114] Ren Z, Zhou P, Tian J (2024). [Plasma long noncoding RNA expression profiles in patients with Parkinson’s disease and the role of lnc-CTSD-5:1 in a PD cell model: a ceRNA microarray-based study]. Nan Fang Yi Ke Da Xue Xue Bao.

[115] Gui Y, Liu H, Zhang L, Lv W, Hu X (2015). Altered microRNA profiles in cerebrospinal fluid exosome in Parkinson disease and Alzheimer disease. Oncotarget.

[116] Wang Q, Han CL, Wang KL, Sui YP, Li ZB, Chen N (2020). Integrated analysis of exosomal lncRNA and mRNA expression profiles reveals the involvement of lnc-MKRN2-42:1 in the pathogenesis of Parkinson’s disease. CNS Neurosci Ther.

[117] Wang H, Lu B, Chen J (2019). Knockdown of lncRNA SNHG1 attenuated Aβ(25–35)-induced neuronal injury via regulating KREMEN1 by acting as a ceRNA of miR-137 in neuronal cells. Biochem Biophys Res Commun.

[118] Wang Q, Ge X, Zhang J, Chen L (2020). Effect of lncRNA WT1-AS regulating WT1 on oxidative stress injury and apoptosis of neurons in Alzheimer’s disease via inhibition of the miR-375/SIX4 axis. Aging (Albany NY).

[119] Bennett CF, Krainer AR, Cleveland DW (2019). Antisense oligonucleotide therapies for neurodegenerative diseases. Annu Rev Neurosci.

[120] Sumner CJ, Miller TM (2024). The expanding application of antisense oligonucleotides to neurodegenerative diseases. J Clin Invest.

[121] Lozupone M, Dibello V, Sardone R, Castellana F, Zupo R, Lampignano L (2023). The development of peptide- and oligonucleotide-based drugs to prevent the formation of abnormal tau in tauopathies. Expert Opin Drug Discov.

[122] McCampbell A, Cole T, Wegener AJ, Tomassy GS, Setnicka A, Farley BJ (2018). Antisense oligonucleotides extend survival and reverse decrement in muscle response in ALS models. J Clin Invest.

[123] Korobeynikov VA, Lyashchenko AK, Blanco-Redondo B, Jafar-Nejad P, Shneider NA (2022). Antisense oligonucleotide silencing of FUS expression as a therapeutic approach in amyotrophic lateral sclerosis. Nat Med.

[124] Deguchi S, Ohka F, Shiba Y, Yamaguchi J, Sato A, Shinjo K (2025). Investigator-initiated phase I trial of an oligonucleotide therapeutic targeting long noncoding RNA TUG1 for recurrent glioblastoma. BMC Cancer.

[125] M SZ, Hartford CCR, Lal A (2021). Interrogating lncRNA functions via CRISPR/Cas systems. RNA Biol.

[126] Gillmore JD, Gane E, Taubel J, Kao J, Fontana M, Maitland ML (2021). CRISPR-Cas9 in vivo gene editing for transthyretin amyloidosis. N Engl J Med.

[127] Lopes D, Lopes J, Pereira-Silva M, Peixoto D, Rabiee N, Veiga F (2023). Bioengineered exosomal-membrane-camouflaged abiotic nanocarriers: neurodegenerative diseases, tissue engineering and regenerative medicine. Mil Med Res.

[128] Wang BP, Pi Y, Chen M, Chang K (2024). [Research progress on exosomes and their non-coding RNAs in the diagnosis of neurodegenerative diseases]. Zhonghua Yu Fang Yi Xue Za Zhi.

[129] Tsakiri M, Tsichlis I, Zivko C, Demetzos C, Mahairaki V (2024). Lipidic nanoparticles, extracellular vesicles and hybrid platforms as advanced medicinal products: future therapeutic prospects for neurodegenerative diseases. Pharmaceutics.

[130] Soto C, Pritzkow S (2018). Protein misfolding, aggregation, and conformational strains in neurodegenerative diseases. Nat Neurosci.

[131] Schoch KM, Miller TM (2017). Antisense oligonucleotides: translation from mouse models to human neurodegenerative diseases. Neuron.

